# Synthesis and Characterization of Allyl Terpene Maleate Monomer

**DOI:** 10.1038/s41598-019-55356-8

**Published:** 2019-12-16

**Authors:** Yan Gu, Matthew Hummel, Kasiviswanathan Muthukumarappan, Zhendong Zhao, Zhengrong Gu

**Affiliations:** 1 Institute of Chemical Industry of Forest Products, CAF, National Engineering Lab. for Biomass Chemical Utilization, Key Lab. of Chemical Engineering of Forest Products, National Forestry and Grassland Administration, Key Lab. of Biomass Energy and Material, Jiangsu Province, Nanjing, 210042 P.R. China; 2 Agricultural & Biosystems Engineering Department, South Dakota State University, Brookings, 57006 USA

**Keywords:** Biotechnology, Chemistry, Engineering, Materials science

## Abstract

Terpenes and their derivatives are sustainable, renewable chemicals that can be used as a complementary hydrocarbon. The exceptions are fossil-based feedstocks and lignin-based feedstocks. A simple method has been found to prepare allyl terpene maleate monomer by substitution reaction at lower reaction temperatures. Using terpenes from turpentine, maleic anhydride and allyl chloride as reactants, the synthesized monomer, terpene-diallyl maleate adduct, was prepared by D-A addition, hydrolysis, and substitution reaction. The resultant monomer was characterized for the first time. The synthesized product will be a versatile monomer and a very important intermediate, having broad application prospects. The synthesized monomer will replace similar aromatic compounds in certain applications because of its low-toxicity and sustainability. The synthesized monomer with two terminal olefin structures has great free radical polymerization potential, according to its physical and chemical properties and exploratory experimentation.

## Introduction

Terpenes and their derivatives are sustainable, renewable chemicals that can be used as a complementary hydrocarbon. The exceptions are fossil-based feedstocks and lignin-based feedstocks^1–4^. Terpenes encompass a large class of terpenoids and are identified as a chain or cyclic olefin, usually grouped according to the number of isoprene (C_5_H_8_)n units in the molecule. Occurring often in plants and marine organisms, terpenes are a natural source of hydrocarbons. Recently, the majority of research has been focused on monoterpenes (C_10_H_16_) primarily produced from turpentine^5–19^.

The authors’ goal is to prepare the synthesized monomer, a terpene maleic acid esters with terminal olefin structure, from turpentine-sourced terpenes. The synthesized monomer will be versatile on its own and also a very important intermediate with broad application prospects. The synthesized monomer will replace similar aromatic compounds in certain applications because of its low-toxicity and sustainability. Due to its special structure, many chemicals could be prepared from the synthesized monomer by addition, oxidation, polymerization, etc. First, the terpene maleate adduct is prepared by isomerization and D-A addition from terpenes, then the synthesized monomer is prepared by subsequent reactions such as esterification or substitution.

In general, the terpene maleate adduct is a simple product that can be industrially produced from terpenes. This terpene maleate adduct can be modified with special structures to be used as epoxy resin curing agents^20,21^, adhesives, insecticide, antiseptic, etc. The terpene maleate adduct can also be used as an intermediate to synthesize a series of important fine chemicals, such as epoxy resin, unsaturated polyester resin, alkyd resin, surfactants, plasticizers, bioactive substances, etc^22–40^.

Several researchers have studied the synthesis of some terpene maleic acid esters. These corresponding terpene maleic acid esters were prepared by the pre-esterification method and following addition or pre-addition followed by esterification. G. M. Wu *et al*. prepared epichlorohydrin and epoxy resin from hydrogenated terpene-maleic anhydride^24^. G. Lai *et al*. prepared Di-2-ethyl hexyl α-pinene-mal eater^28^. Y. Q. Gao *et al*. prepared maleic acid ester by esterification from maleic anhydride and alcohol, then prepared corresponding terpene maleic acid ester by D-A addition reaction from maleic acid ester and β-pinene^34^. J. W. Lian *et al*. prepared bis(2-ethylhexyl)-4-(4-methylpent-3-enyl) cyclohex-4-ene-1,2- dicarboxylate by D-A addition reaction and esterification reaction from β-myrcene^38^. K. Huang *et al*. prepared terpene-dimethyl maleate adducts by D-A addition reaction from industrial dipentene and dimethyl maleate under atmospheric and pressurized conditions^40^. The authors wanted to directly introduce a terminal olefin structure into terpene maleate adduct. Following which, the target synthesized monomer could be prepared. The selectivity and yield of corresponding terpene maleic acid esters with terminal olefin structure were low using the above esterification method^24,28,34,38,40^. This is due to the relatively large steric hindrance of the bicyclic structure of the terpene maleate adduct and the instability of the alcohol and halogenated hydrocarbons with terminal olefin structure at high reaction temperature. The majority of researchers avoided directly introducing the terminal olefin structure into the terpene maleate adduct. Only in 1945, Cottrell, Armitage and Hewitt mentioned the synthesis and application of terpene-diallyl maleate adduct^41^, but they didn’t characterize and identify it. After Cottrell, Armitage and Hewitt’s patent, there were no publications on the synthesis, identification, characterization, or application of the terpene-diallyl maleate adduct.

In the authors’ study, a simple method was found to prepare the versatile allyl terpene maleate monomers^42–45^. The terpene maleate adduct was prepared from terpene. Then, terpene-diallyl maleate adduct was, for the first time, prepared by a hydrolysis reaction and substitution reaction from terpene maleate adduct at lower reaction temperatures. The effects of reaction time, reaction temperature, molar ratio, catalyst amount, and acid binding agent amount on the yield were investigated. The synthesized monomer was characterized and identified by GC-MS, FT-IR, powder XRD, TG-DTA, ^1^H NMR and ^13^C NMR and the exploratory experiment of UV curing was carried out.

## Experimental

### Materials

All the reagents used in this work were of analytical grade.

### Synthesis of terpene-diallyl maleate adduct

Terpene maleate adduct was prepared according to ref. ^46^. 49 g (0.5 mol) maleic anhydride was added into a 250 ml four necked flask equipped with a electric agitator, a thermometer, a reflux condenser, and a dropping funnel. Slowly heating (5 °C/min) until maleic anhydride was completely melted, then continuous heated up to 145 °C. 0.96 g (0.006 mol) p-toluene sulfonic acid as catalyst was added into the above four necked flask. Then 96 g (0.71 mol) α-pinene, β-pinene, dipentene, terpinolene or α-terpinene was dropped into the above four necked flask in 0.5 hour. The reaction was completed at 145 °C for 1.5–2 hours. After reaction, the pure terpene maleic adduct was obtained after extraction with distilled water for 2–3 times, followed by distillation under vacuum of 0.096–0.098 Mpa for removing the unreacted terpene.

**Figure 1:**
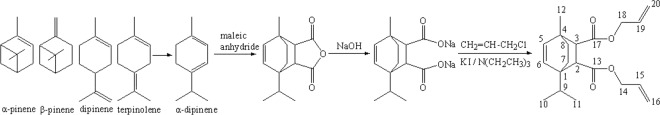
Synthesis of terpene-diallyl maleate adduct.

Terpene maleate sodium salt was prepared by alkalized terpene maleate adduct with sodium hydroxide (same molar amount) in water for 2 hours at 60 °C under magnetic stirring followed with drying. If not specified, 2.96 g (0.01 mol) terpene maleate sodium salt, 4.59 g (0.06 mol) allyl chloride, 0.66 g (0.004 mol) catalyst e.g. KI, 3.03 g (0.03 mol) acid binding agent e.g. triethylamine, a trace of p-benzoquinone as a polymerization inhibitor and 20–30 ml solvent DMF were added into a 100 ml three-necked flask equipped with a thermometer, a reflux condenser, and a dropping funnel. Then the reaction was conducted at 60 °C for 16 hours, depending on the treatment. When the reaction was completed, the reaction solution was extracted with n-hexane and the synthesized monomer was obtained after the evaporation of n-hexane. The synthesis formula of terpene-diallyl maleate adduct is shown in Fig. 1.

### UV curing exploratory experiment of the synthesized monomer

1 gram synthesized monomer, 2 times diluents (tetrahydrofuran) and 5% photoinitiator (2,2-Dimethoxy-2-phenylacetophenone) were weighed and mixed well. Then, the above solution was coated on glass sheet and irradiated with a 160 W mercury lamp (365 nm) for about 30 minutes. The UV cured product was characterized by FT-IR, powder XRD and TG-DTA.

### Analysis methods

The general gas chromatography-mass spectrometry profile of terpene-diallyl maleate adduct was obtained on a GC-MS system (Shimadzu GCMS-QP2010EP) equipped with Rxi-5Sil MS quartz capillary column (length 30.0 m, inner diameter 0.25 mm, film thickness 0.25 μm, injection volume 2.0 μL). The column was held at 130 °C for 0 min, and then heated to 240 °C at a rate of 8 °C/min for 5 min. Identification of the components of terpene-diallyl maleate adduct was confirmed using total ion chromatograms and the fragmentation pattern. Fourier transform infrared spectroscopy (FT-IR) analysis of the terpene maleate adduct, terpene maleate sodium salt and terpene-diallyl maleate adduct was performed on a FT-IR Spectrometer (Spectrum Two FT-IR Spectrometer, resolution: 0.5 cm^−1^, scan: 20 times, range: 4000–500 cm^−1^, thin film, 25 °C).

^1^H NMR and ^13^C NMR analysis were conducted with Bruker nuclear magnetic resonance instrument at 600 MHz and 150 MHz respectively. CDCl_3_ was used as the solvent.

## Results and Discussion

### Effects of reaction parameters on the substitution reaction

**Figure 2:**
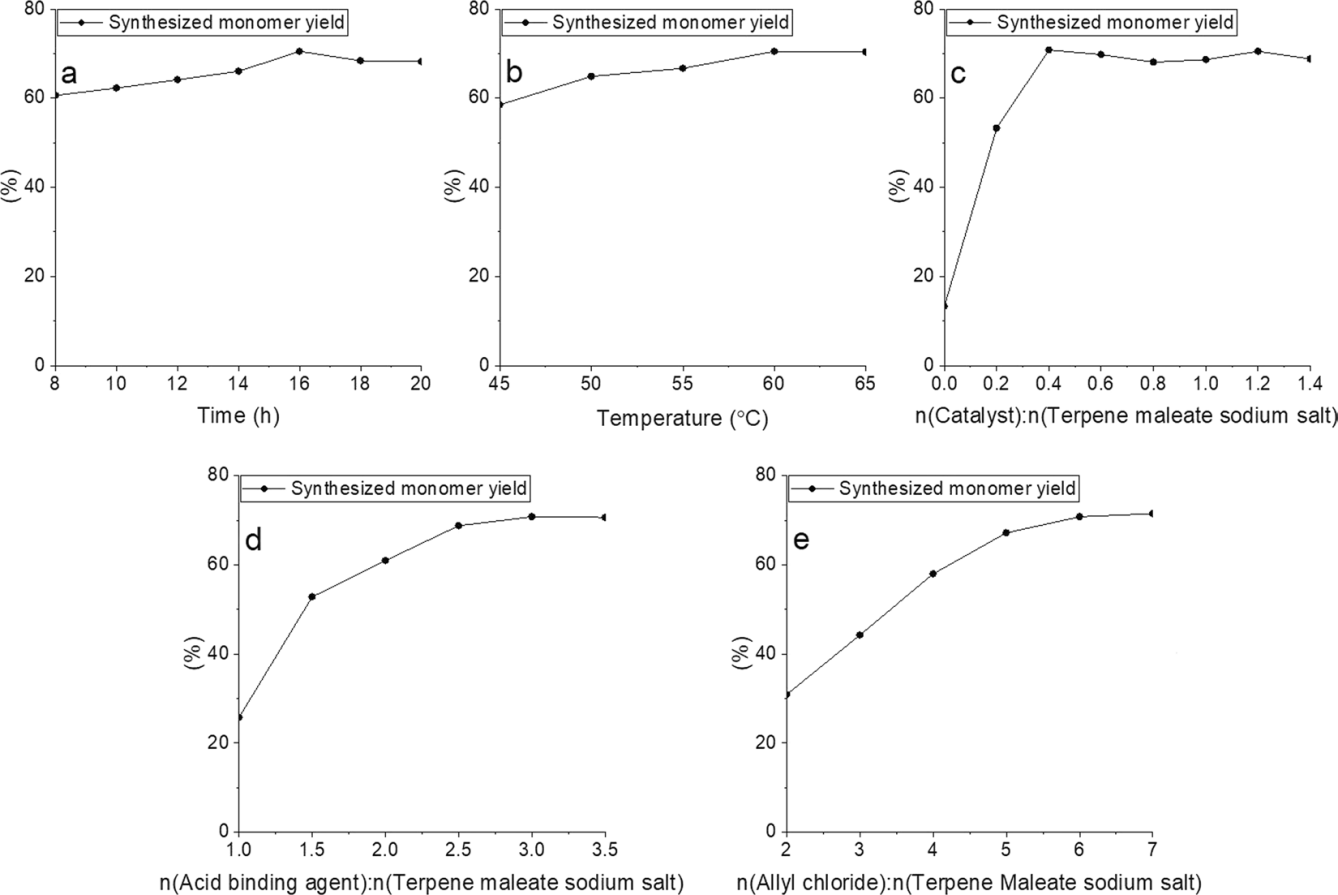
Effects of reaction parameters on the substitution reaction (**a**) the effect of reaction time; (**b)** the effect of reaction temperature; (**c**) the effect of catalyst; (**d**) the effect of acid binding agent; (**e**) the effect of the ratio of n(Allyl chloride):n(Terpene maleate sodium salt)). Reaction conditions^1*^: Without specification, the reaction conditions were listed as follows: reaction temperature 60 °C, reaction time 16 hours, the molar ratio of n(Catalyst):n(Terpene maleate sodium salt) was 0.4:1, the molar ratio of n(Acid binding agent):n(Terpene maleate sodium salt) was 3:1, the molar ratio of n(Allyl chloride):n(Terpene maleate sodium salt) was 6:1 and trace p-benzoquinone as a polymerization inhibitor.

The substitution reaction was an S_N_2 reaction. The intermediate transition state was formed and then the synthesized monomer was obtained. The formation rate of the intermediate transition state determined the substitution reaction rate and was affected by the reaction activity of halogenated hydrocarbons, nucleophilicity of terpene maleate sodium salt, the steric hindrance, and the solubility of reactants in the solvent. The effects of the reaction factors, such as reaction time, reaction temperature, catalyst amount, acid binding agent amount, and molar ratio, were investigated for their impact on the synthesized monomer yield. The results of which are shown in Fig. 2.

The effect of reaction time was investigated and is shown in Fig. 2a. The reaction conditions were identical to reaction conditions^1*^ except for the ratio of n(Catalyst): n(Terpene maleate sodium salt) at 1.2:1. The synthesized monomer yield increased from 60.63% at 8 hours to 70.47% at 16 hours, then decreased to 68.21% at 20 hours. The S_N_2 reaction was dominant in the first 16 hours, then a slight polymerization reaction occurred after 16 hours. According to the result, the optimum reaction time was selected to be 16 hours.

The effect of reaction temperature was investigated and is shown in Fig. 2b. The reaction conditions were the same as the reaction conditions^1*^ except the ratio of n(Catalyst): n(Terpene maleate sodium salt) was at 1.2:1. The suitable reaction temperature was in the range of 45–65 °C, according to the boiling point of allyl chloride. The synthesized monomer yield increased from 58.52% at 45 °C to 70.47 at 60 °C, then gradually plateaued with the increase of reaction temperature. The reaction rate was greatly affected by the reaction temperature when the reaction temperature was low. The reaction rate was less affected by reaction temperature when the reaction temperature reached 60 °C. According to these results, the optimum reaction temperature was selected to be 60 °C.

The effect of the catalyst dosage was investigated and is shown in Fig. 2c. The reaction conditions were the same as the reaction conditions^1*^. The synthesized monomer yield increased significantly from 13.27% without the catalyst to 70.78% when the ratio of n(Catalyst): n(Terpene maleate sodium salt) was 0.4:1; however further increasing the catalyst dosage did not improve yield. In general, the reactivity of halogen was in the expected order: I > Br > Cl^44^. The S_N_2 reaction occurred easily because chlorine in allyl chloride could be replaced by iodine in KI and generated allyl iodide with higher reactivity Iodine released from KI was in a state of substitution and reduction during the S_N_2 reaction. Allyl iodide was not used directly because of its instability and high price. According to the results, the optimum molar ratio n(Catalyst):n(Terpene maleate sodium salt) was selected to be 0.4:1.

The effect of the acid binding agent was investigated and is shown in Fig. 2d. The reaction conditions were the same as the reaction conditions^1*^. The synthesized monomer yield increased from 25.78%, when the ratio of n(Acid binding agent):n(Terpene maleate sodium salt) was 1:1, to 70.78%, when the ratio of n(Acid binding agent):n(Terpene maleate sodium salt) was 1:3 and then gradually plateaued with the increase of the acid binding agent. The acid binding agent could be used similarly as a phase transfer catalyst. First, a quaternary ammonium salt was generated with allyl iodide generated from allyl chloride, then the intermedia was generated by nucleophilic substitution with terpene maleate sodium salt. Finally, terpene-diallyl maleate adduct was generated and the acid binding agent was released^42–45^. According to the result, the optimum molar ratio of n(Acid binding agent):n(Terpene maleate sodium salt) was selected to be 3:1.

The effect of the ratio of n(Allyl chloride):n(Terpene maleate sodium salt) was investigated and is shown in Fig. 2e. The reaction conditions were the same as the reaction conditions^1*^. The synthesized monomer yield increased from 30.94% when the ratio of n(Allyl chloride): n(Terpene maleate sodium salt) was 2:1 to 70.78% when the ratio of n(Allyl chloride): n(Terpene maleate sodium salt) was 6:1 and then gradually plateaued with the increase of allyl chloride. According to the result, the optimum ratio of n(Allyl chloride): n(Terpene maleate sodium salt) was selected to be 6:1.

The optimum reaction conditions were a reaction temperature 60 °C and a reaction time of 16 hours, the ratio of n(Catalyst):n(Terpene maleate sodium salt) at 0.4:1, the ratio of n(Acid binding agent):n(Terpene maleate sodium salt) at 3:1, the ratio of n(Allyl chloride): n(Terpene maleate sodium salt) at 6:1 and trace p-benzoquinone as a polymerization inhibitor. The results of repeat experiments are shown in Table 1. Herein, we used DMF as a solvent, even with a recognized environmental concern, because other available solvents such as hexane and acetone did not achieve satisfactory monomer yield (Table S1).

**Table 1:** The results of repeat experiments.

Trial	Synthesized monomer yield/%
1	70.78
2	69.76
3	71.25

**Figure 3:**
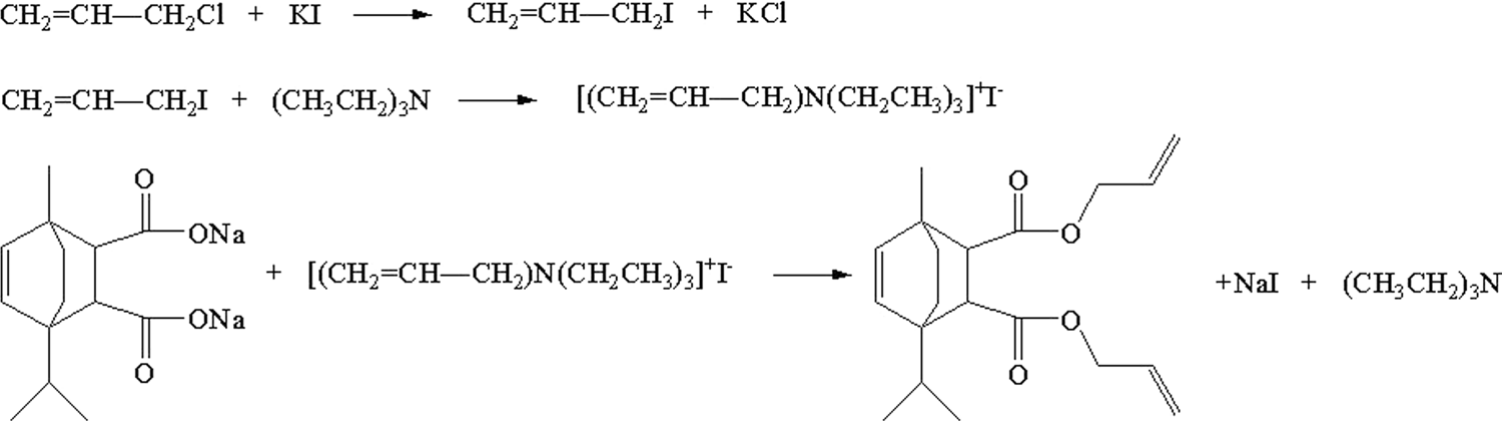
Mechanism of the S_N_2 substitution reaction.

The substitution reaction was S_N_2 reaction. Chlorine in allyl chloride was replaced by iodine in KI and allyl iodide with higher reactivity was generated. Then, allyl iodide reacted with acid binding agent and the intermediate was formed. The intermediate reacted with terpene maleate sodium salt and the synthesized monomer was obtained. The mechanism might be the following (Fig. 3).

### Characterization of terpene-diallyl maleate adduct

**Figure 4:**
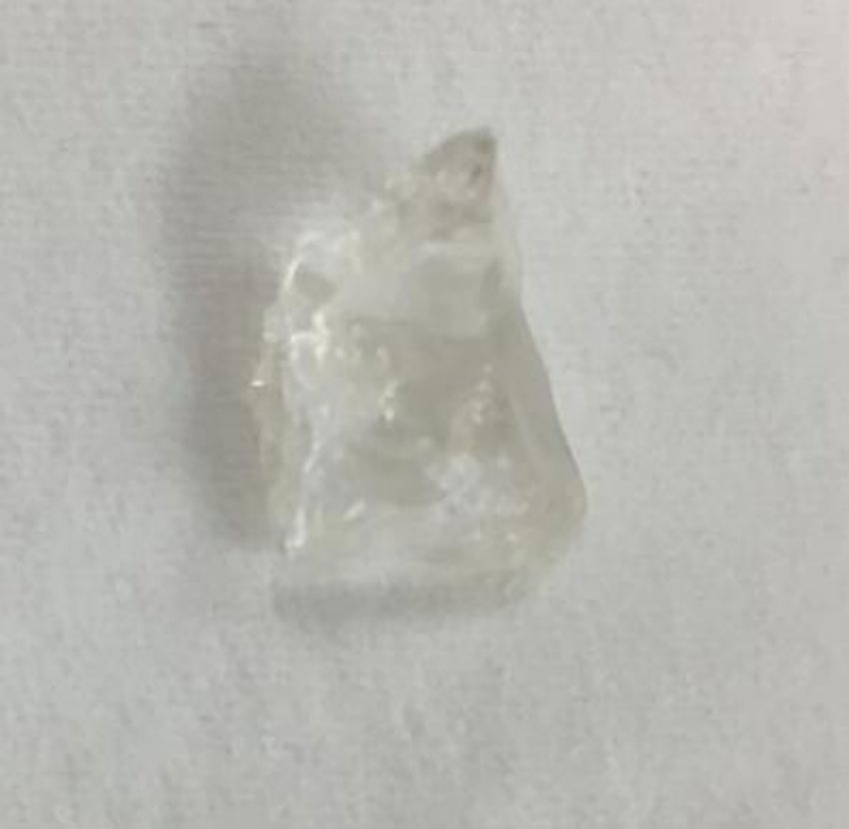
Crystal of terpene-diallyl maleate adduct.

**Figure 5:**
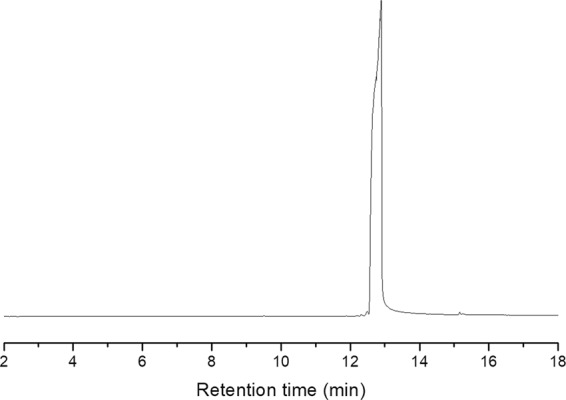
Gas chromatograms of terpene-diallyl maleate adduct.

The purified synthesized monomer, terpene-diallyl maleate adduct, was an almost colorless crystal (Fig. 4) and only showed 1 peak in the gas chromatogram profile (Fig. 5). The content of terpene-diallyl maleate adduct is 98%.

**Figure 6:**
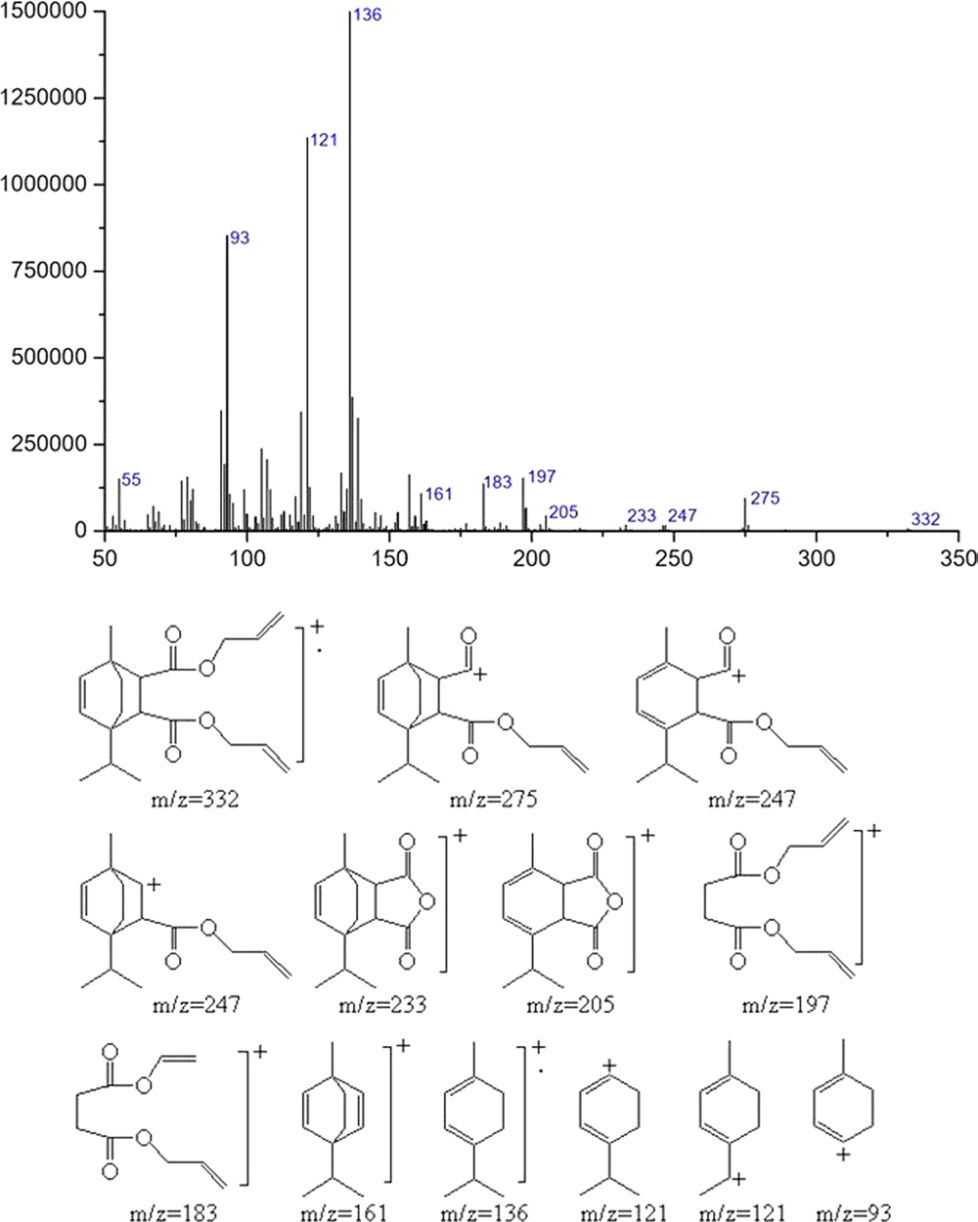
Mass spectra and the possible fragment ions of terpene-diallyl maleate adduct.

GC-MS was used to identify the synthesized monomer. The mass spectra and the possible fragment ions of terpene-diallyl maleate adduct are shown in Fig. 6. In Fig. 6, m/z 332 was the molecular ion peak of terpene-diallyl maleate adduct, and the fragment peak of m/z 275 was [M-OCH_2_CHCH_2_]^+^ formed by removing one molecular ester moiety. The peak of m/z 233 was a stable terpene maleic anhydride structure formed by de-esterification, and the base peak of 136 was a stable terpene skeleton structure, and the generation of small mass ions in the mass spectrum was random.

**Figure 7:**
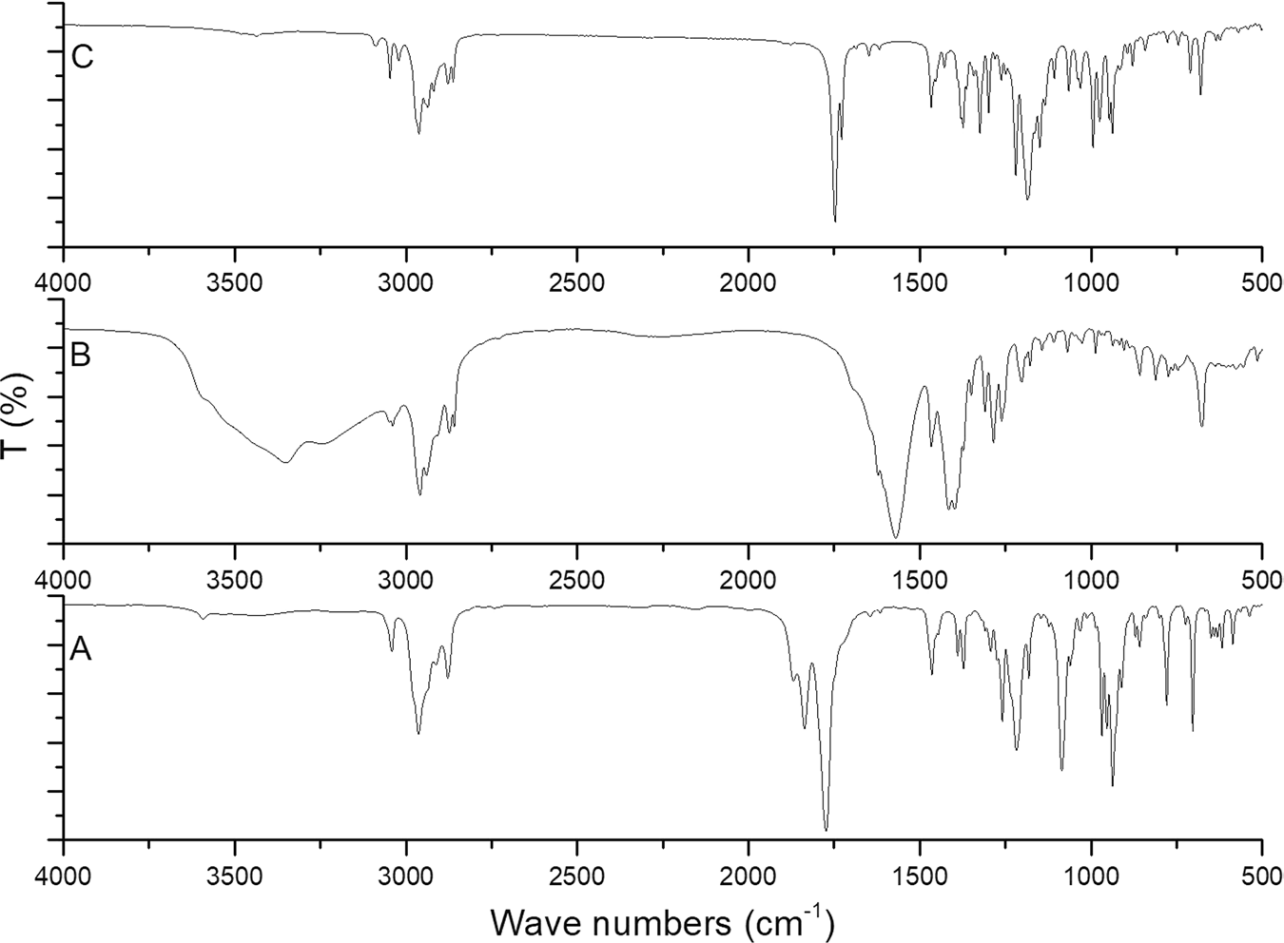
FT-IR spectra of terpene-diallyl maleate adduct, terpene maleate sodium salt and terpene maleate adduct ((**A**) terpene maleate adduct; (**B**) terpene maleate sodium salt; (**C**) terpene-diallyl maleate adduct).

FT-IR spectrum of terpene maleate adduct, terpene maleate sodium salt and terpene-diallyl maleate adduct are shown in Fig. 7. The main characteristic absorption peaks of terpene maleate adduct were the double peaks generated by the C=O coupling vibration of the anhydride at 1834 and 1774 cm^−1^, and the low wave number peak was slightly stronger, which was a typical characteristic of cyclic anhydride. The stretching vibration absorption peaks of methyl and methylene groups were at 2962, 2936, 2876, and 2862 cm^−1^. The C-H bending vibration absorption peaks was at 1466 and 1373 cm^−1^. The stretching vibration absorption peak of C-O was at 1084 cm^−1^. The stretching vibration absorption peak of the double bond C-H at the ring was at 3040 cm^−1^ and the stretching vibration absorption peak of double bond C=C was extremely weak at 1645 cm^−1^. After becoming salt, the peaks of C=O of anhydride at 1834 and 1774 cm^−1^ disappeared. Only the peaks of C=O of salt was visible at 1570 and 1466 cm^−1^, which was the symmetric and antisymmetric stretching vibration absorption peaks of the carboxylate (–CO_2_–).

The main absorption peaks of terpene-diallyl maleate adduct was at 2962, 2876, 1747, 1466, 1373 and 1186 cm^−1^. The stretching vibration absorption peaks of methyl and methylene groups were at 2962, 2936, 2876, and 2862 cm^−1^. The C-H bending vibration absorption peaks was at 1466 and 1373 cm^−1^. Terpene-diallyl maleate adduct was different from terpene maleate adduct and terpene maleate sodium salt. The C=O peaks of anhydride and salt disappeared, and only the stretching vibration absorption peaks of C=O of ester was at 1747 and 1727 cm^−1^ (the product was crystallized after purification, which was caused by different crystal structure (Fig. S1)). The stretching vibration absorption peak of C-O was at 1186 cm^−1^. The vibration absorption peaks of C-H of the terminal olefin structure were at 3088 and 3021 cm^−1^. The stretching vibration absorption peak of C-H of the double bond in the ring was at 3040 cm^−1^. The stretching vibration absorption peak of the C=C, which was at 1648 cm^−1^, was significantly enhanced compared with terpene maleate adduct.

**Figure 8:**
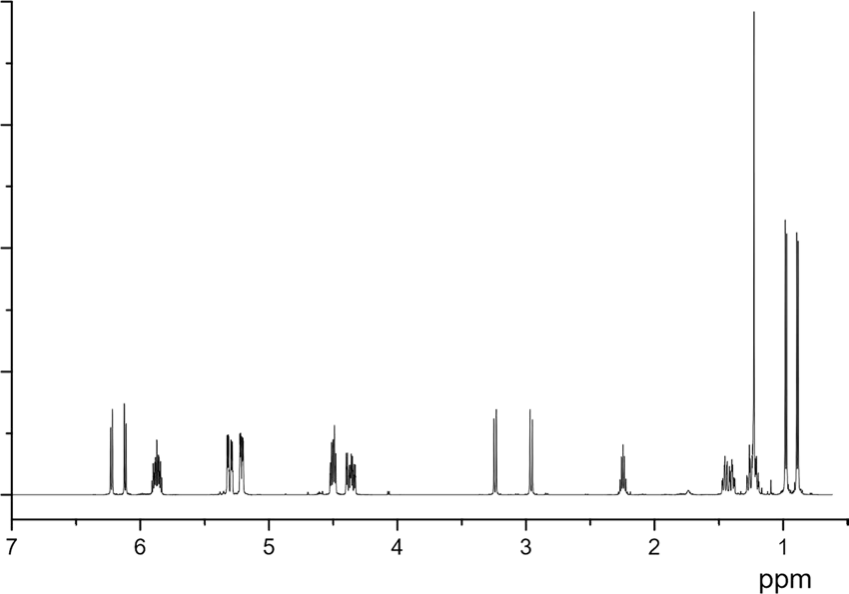
^1^H NMR spectrum of terpene-diallyl maleate adduct.

Figure 8 shows the ^1^H NMR spectrum of terpene-diallyl maleate adduct. The specific analysis was as follows: δ: 2.96(d, 1H, C2—H), 3.24(d, 1H, C3—H), 6.22(d, 1H, C5—H), 6.12 (d, 1H, C6—H), 1.47–1.19(m, 4H, C7—H, C8—H), 2.24(m, 1H, C9—H), 0.88(d, 3H, C10—H), 0.98(d, 3H, C11—H), 1.23(s, 3H, C12—H), 4.50–4.36(m, 4h, C14—H, C18—H), 5.87(m, 2H, C15—H, C19-H), 5.22 and 5.20(d, 2H, C16—H), 5.32 and 5.29(d, 2H, C20—H).

**Figure 9:**
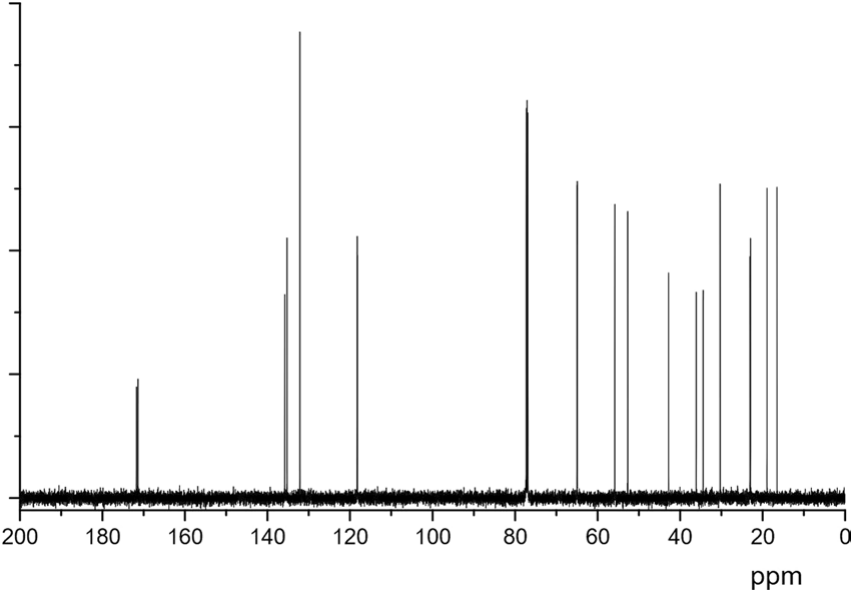
^13^C NMR spectrum of terpene-diallyl maleate adduct.

Figure 9 shows the ^13^C NMR spectrum of terpene-diallyl maleate adduct. It was the peaks of solvent at δ 77.3, 77.1 and 76.9. δ 171.7 and 171.4 were chemical shifts of C=O, δ 132.1 was the chemical shift of C15 and C19, and δ 118.2 and 118.3 were the chemical shifts of C16 and C20. The chemical shifts of carbon atoms and the corresponding proton are shown in Table 2.

**Table 2:** ^13^C NMR chemical shift of each carbon atom from terpene-diallyl maleate adduct.

Carbon number	δ (C)	δ (H)	Carbon number	δ (C)	δ (H)
1 C	42.8	—	11 CH_3_	22.9	0.98
2 CH	52.7	2.96	12 CH_3_	16.5	1.23
3 CH	55.8	3.24	13 C	171.4	—
4 C	34.4	—	14 CH_2_	64.9	4.50–4.36
5 CH	135.8	6.22	15 CH	132.1	5.87
6 CH	135.3	6.12	16 CH_2_	118.2	5.22–5.20
7 CH_2_	18.9	1.47–1.19	17 C	171.7	—
8 CH_2_	36.1	1.47–1.19	18 CH_2_	65.0	4.50–4.36
9 CH	30.3	2.24	19 CH	132.1	5.87
10 CH_3_	23.0	0.88	20 CH_2_	118.3	5.32–5.29

**Figure 10:**
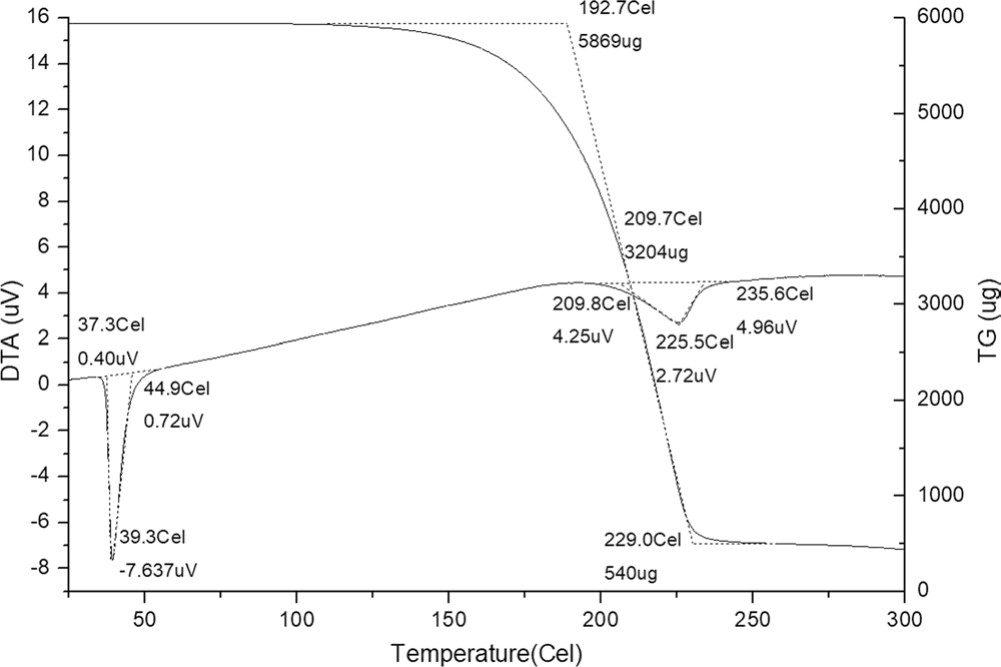
TG-DTA of terpene-diallyl maleate adduct.

The melting point and boiling point of terpene-diallyl maleate adduct was determined by TG-DTA (Fig. 10). The melting point was 37.3 °C and the boiling point was 192.7 °C.

**Figure 11:**
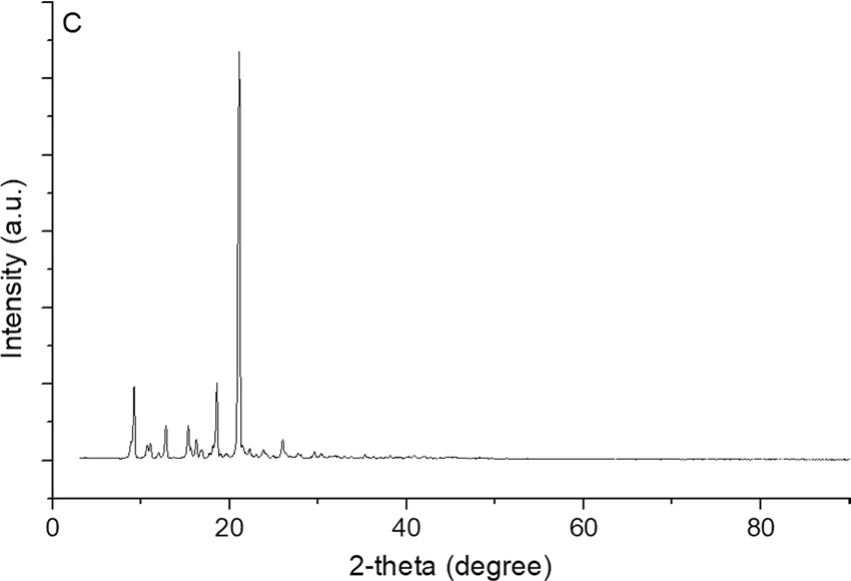
XRD of terpene-diallyl maleate adduct (C: terpene-diallyl maleate adduct).

The XRD results of terpene-diallyl maleate adduct (Fig. 11) indicated a defined crystalline structure. The peak was the largest at 21.2°, and there were other peaks in the range of 9.2 to 25.0°, which indicated that it has a variety of crystalline structures.

### Exploratory study on UV curing properties of terpene-diallyl

#### Maleate adduct

**Figure 12:**
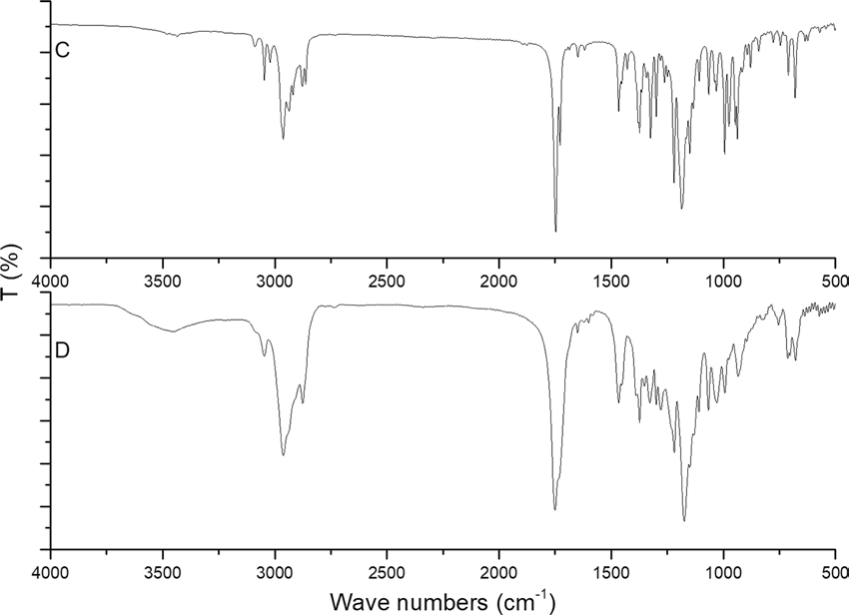
FT-IR spectra of terpene-diallyl maleate adduct and its cured product (C: terpene-diallyl maleate adduct; D: cured products).

According to the structure identification and characterization results (NMR, FT-IR and GC-MS) of terpene-diallyl maleate adduct, there were two terminal olefin structures which make the new monomer a suitable feedstock of free radical polymerization. The exploratory UV curing experiment was carried out in order to determine whether it had sufficient reactive functional groups and could be used as a free radical polymerizable monomer. The terpene-diallyl maleate adduct and its cured product was characterized by FT-IR and the FT-IR spectrum of them is shown in Fig. 12.

Before curing, the obvious vibration absorption peaks at 3088 and 3021 cm^−1^ and obvious stretching vibration absorption peaks of C=C double bonds at 1648 cm^−1^, indicated terminal olefin structure C-H. In addition, the stretching vibration absorption peaks of C=O at 1747 and 1727 cm^−1^ was possibly caused by the different crystal structure of the monomer. After UV curing, the obvious vibration absorption peaks of the terminal olefin structure C-H at 3088 and 3021 cm^−1^ disappeared, and the stretching vibration absorption peak of C=C double bond at 1648 cm^−1^ was significantly weakened. There was only one stretching vibration absorption peak of C=O at 1747 cm^−1^ and the peak at 1727 cm^−1^ disappeared because there was no crystal structure difference in the cured product. The result demonstrated that terpene-diallyl maleate adduct could undergo free radical polymerization under UV radiation. Furthermore, the UV-cured product demonstrated better thermochemical stability, its initial decomposition temperature was 263.4 °C (Fig. S2). For details, the double bond in the terminal olefin structure was opened and polymerization occurred, and the double bond in the ring was not reacted.

**Figure 13:**
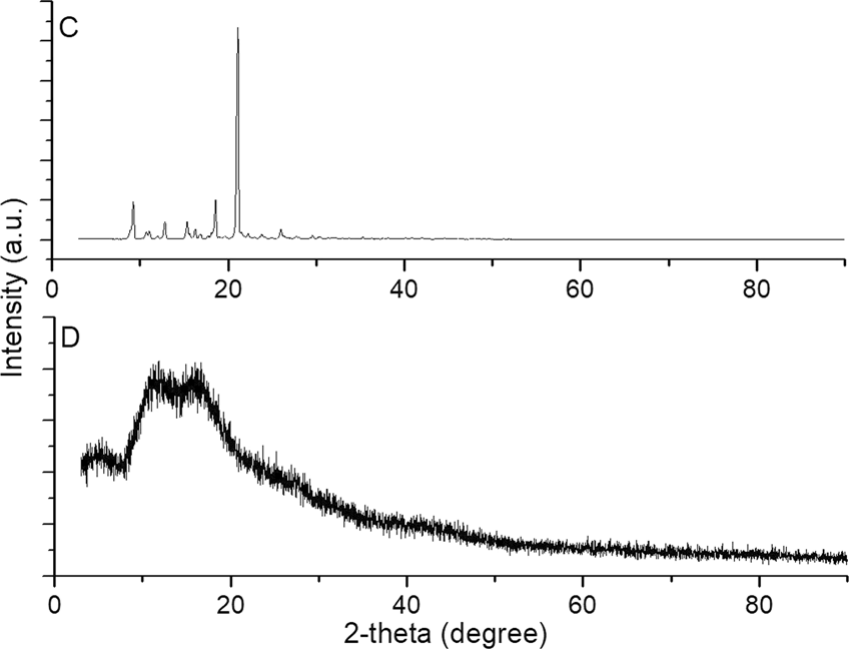
XRD of terpene-diallyl maleate adduct and its cured product (C: terpene-diallyl maleate adduct; D: cured products).

The XRD results of terpene-diallyl maleate adduct and its cured product (Fig. 13) indicated that terpene diallyl maleate was a defined crystalline structure, and its cured products were amorphous, non-crystalline structure. There were significantly different from terpene diallyl maleate and its cured products. The XRD results of terpene-diallyl maleate adduct and its cured products indicated that the original crystalline structure of terpene-diallyl maleate adduct was destroyed while it was polymerized under UV irradiation.

The FT-IR spectrum and XRD results of terpene-diallyl maleate adduct and its cured product proved that terpene-diallyl maleate adduct had sufficient reactive functional groups and could be used as a free radical polymerizable monomer.

## Conclusions

A simple method was found to prepare allyl terpene maleate monomer by substitution reaction at lower reaction temperature. The synthesized monomer, terpene-diallyl maleate adduct, with two-terminal olefin structures was prepared and the optimum reaction conditions to prepare the synthesized monomer were obtained. The results of the synthesized monomer characterization indicated that it was the target synthesized monomer and had two terminal olefins in its structure. The synthesized monomer will be a versatile monomer and a very important intermediate with broad application prospects. The UV curing exploratory experiment was carried out, and the result proved that the synthesized monomer had sufficient reactive functional groups and could be used as a free radical polymerizable monomer. The preparation, application, and mechanism of the polymers from the synthesized monomer are for further study. The other applications are also to be studied further.

## Supplementary information

Supplementary information
