# Supplementary material for: Synthesis and Characterization of Allyl Terpene Maleate Monomer
Source: Sci Rep. 2019 Dec 16;9:19149. doi: 10.1038/s41598-019-55356-8 (PMC6914792; doi:10.1038/s41598-019-55356-8)
Supplement: Supplementary file 1 — Supplementary information [file 41598_2019_55356_MOESM1_ESM.doc]

Supplementary

Figure S1 indicated that terpene-diallyl maleate adduct had different crystal structure.


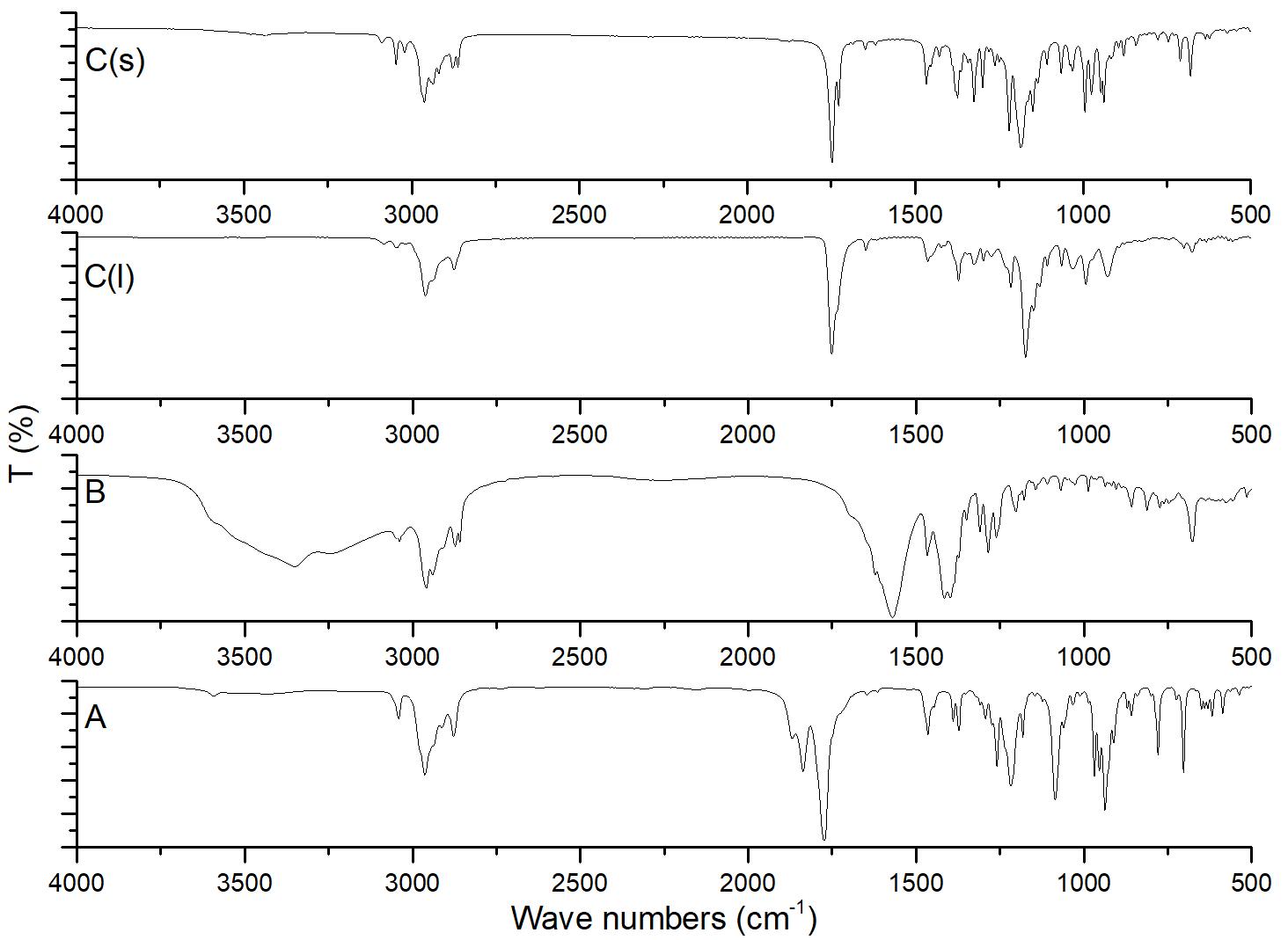
The results of synthesized monomer yield with different solvents are shown in Table S1. We used DMF as a solvent, even with a recognized environmental concern, because other regular solvents such as hexane and acetone did not achieve satisfactory monomer yield.

Figure S1 FT-IR spectra of terpene-diallyl maleate adduct, terpene maleate sodium salt and terpene maleate adduct (A: terpene maleate adduct; B: terpene maleate sodium salt; C(l): terpene-diallyl maleate adduct (sample solution); C(s): terpene-diallyl maleate adduct (solid sample))

Table S1 Synthesized monomer yield with different solvents

| Solvent | Synthesized monomer yield / % |
| --- | --- |
| DMF | 70.78 |
| Hexanes | 2.91 |
| Actone | 32.41 |

Figure S2 indicated that terpene-diallyl maleate adduct was cured in the presence of photoinitiator under UV light conditions. The cured products were different from terpene-diallyl maleate adduct according to the TG-DTA (Figure S2). Initial decomposition temperature of cured products was 263.4 ℃.


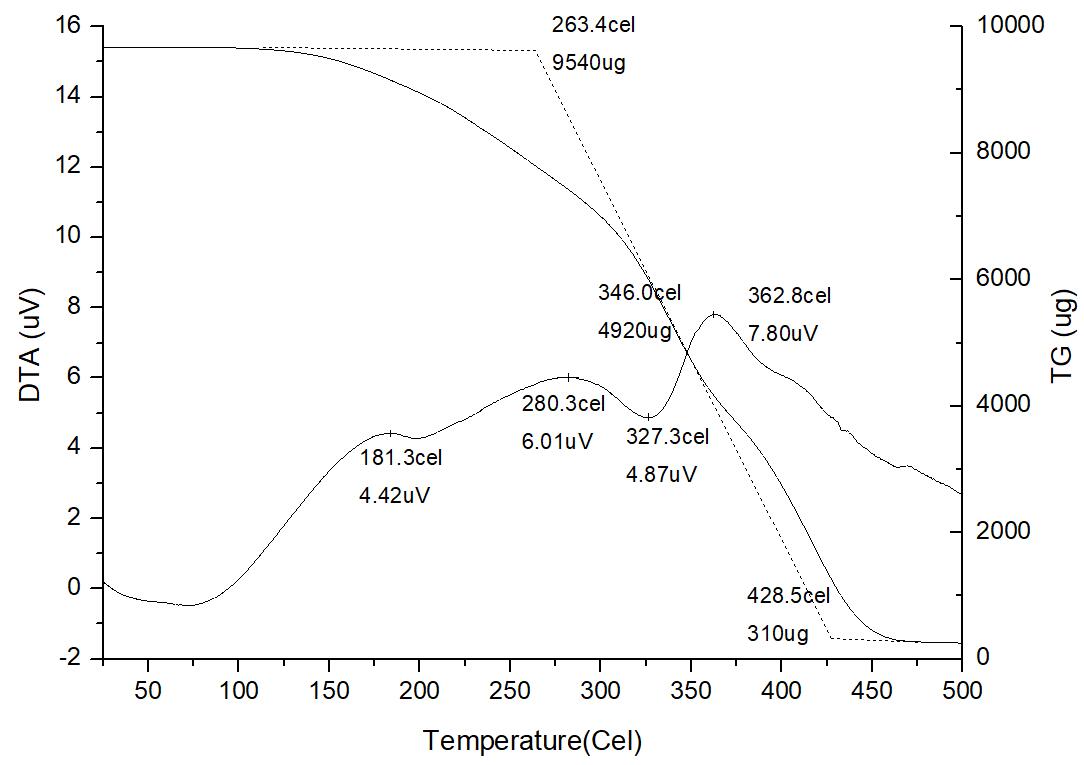


Figure S2 TG-DTA of cured products
